# Presentation, Treatment, and Outcome of a First Stroke-like Episode in a Carrier of the Compound Heterozygous Variants c.695G_A and c.2209G_C in POLG: A Case Report

**DOI:** 10.7759/cureus.78428

**Published:** 2025-02-03

**Authors:** Dominik Zieglgänsberger, Josef Finsterer

**Affiliations:** 1 Neurology, Kantonshospital St. Gallen, St. Gallen, CHE; 2 Neurology, Neurology and Neurophysiology Center, Vienna, AUT

**Keywords:** antiepileptic drugs, epilepsy, hereditary neuropathy, polg1, stroke-like episode

## Abstract

In this report, we describe the case of a 19-year-old female patient with two heterozygous *POLG* variants in different exons manifesting phenotypically as polyneuropathy and later with a first stroke-like episode (SLE) and seizures. This has not been reported earlier. The patient suffered from *POLG*-related neuropathy since the age of 14 and was admitted for her first SLE, which manifested clinically as hemianopia and two days later led to impaired consciousness, hemiparesis, seizures, and status epilepticus. Cerebral MRI showed a corresponding stroke-like lesion (SLL) in the right occipital lobe. The seizures were treated with levetiracetam, lacosamide, and perampanel, but the treatment of status epilepticus required anesthesia with thiopental, ketamine, and midazolam. In addition, glucocorticoids, nitric oxide (NO) precursors, and antioxidants were administered. Under this treatment, the SLL initially increased in size and intensity before regressing. After extubation, recurrent focal motor status epilepticus occurred, which could be stopped with midazolam. This case shows that *POLG* mutations can initially manifest phenotypically with polyneuropathy and SLE, which occurs before the onset of seizures, status epilepticus due to *POLG* variants may require thiopental anesthesia and that levetiracetam, lacosamide, and perampanel may have a favorable long-term effect on seizure activity in carriers of *POLG *mutations.

## Introduction

Mitochondrial disorders (MIDs) are not only caused by mutations in mtDNA but also by those in nuclear DNA. One of the nuclear genes that are frequently mutated in MIDs is *POLG*. *POLG* variants present with heterogeneous phenotypes known as *POLG* spectrum disorders, ranging in severity from asymptomatic or mild to fatal [1]. A rare clinical manifestation of *POLG* variants is hereditary neuropathy, which often manifests together with dysarthria/dysphagia and ophthalmoplegia (sensory ataxic neuropathy, dysarthria, and ophthalmoparesis (SANDO) syndrome) [2]. Much more frequently than in the peripheral nervous system (PNS), *POLG* variants manifest phenotypically in the central nervous system (CNS) [3]. *POLG* phenotypes with CNS manifestations include Leigh syndrome, MELAS (mitochondrial encephalopathy, lactic acidosis, and stroke-like episodes) syndrome, Alpers-Huttenlocher syndrome (AHS), also known as progressive sclerosing poliodystrophy, and MNGIE (mitochondrial neuro-gastro-intestinal encephalopathy) syndrome, mitochondrial depletion syndrome (MDS), mitochondrial recessive ataxia syndrome (MIRAS), spinocerebellar ataxia with epilepsy (SCAE), myoclonic epilepsy, myopathy and spinocerebellar ataxia (MEMSA), isolated epilepsy, Parkinson's syndrome, dementia, developmental delay, and rare stroke-like episodes (SLEs) [3-6]. *POLG*-related disease is diagnosed based on clinical presentation, cerebral imaging, EEG, and evidence of a pathogenic *POLG* variant.

The *POLG* gene is located on the long arm of chromosome 15 in position 26.1 and has 23 exons. It produces a 140 kDa protein consisting of 1239 amino acids [7]. The protein encoded by *POLG* belongs to the family of type A DNA polymerases. It is a mitochondrial nucleoid with a Mg2+ cofactor and 15 turns, 52 beta-strands, and 39 alpha-helixes [7]. POLG contains a polyglutamine tract near its N-terminus, which may be polymorphic. Two transcript variants encoding the same protein have been found for this gene: *POLG1* and *POLG2* [7]. POLG is the only known DNA polymerase in human mitochondria and is essential for mitochondrial DNA replication and repair [8]. It is known that defects in mtDNA replication lead to mitochondrial dysfunction and disease. Several hundred coding variations have been identified in the gene encoding the catalytic subunit of *POLG* [8]. However, a patient carrying two heterozygous *POLG* variants in different exons, phenotypically manifesting initially as polyneuropathy and later with an SLE and seizures, has not yet been described.

## Case presentation

The index patient was a 19-year-old woman with *POLG*-related MID due to c.695G>A and c.2209G>C variants, who initially manifested with neuropathy at the age of 14 years. The neuropathy, which manifested clinically with sensory disturbances and exercise intolerance, was classified as axonal sensorimotor polyneuropathy based on nerve conduction studies and required the use of a wheelchair for long distances (Table 1). At 19 years of age, the patient had the first SLE with the initial clinical manifestation being hemianopia to the left on waking in the morning, which led to hospitalization (hospital day (HD) 1).

**Table 1:** Timeline of the disease course BSP: burst suppression pattern; SLE: stroke-like episode; SLL: stroke-like lesion

Age (years)	Hospital Day	Diagnosis/event	Treatment
14		axonal neuropathy	wheelchair for long distances
19	1	SLE (hemianopia), epileptiform discharges	LEV
19	3	hemiparesis, status epilepticus	LEV, LCM, PER
19	4	status epilepticus	midazolam
19	5	status epilepticus	midazolam increased
19	6	status epilepticus, BSP	thiopental, ketamine
19	7	BSP	thiopental increased
19	8	BSP	thiopental reduced
19	9	BSP	thiopental reduced
19	10	BSP	thiopental reduced
19	12	BSP, rhythmic delta	thiopental withdrawn
19	13	regression of SLL	ketamine reduced
19	14	none	ketamine reduced
19	15	spiky EEG	ketamine reduced
19	17	none	ketamine withdrawn
19	18	none	midazolam reduced
19	19	none	midazolam reduced
19	21	none	midazolam withdrawn
19	27	further regression of SLL	none
19	28	tracheotomy, recurrent focal status	midazolam
19	52	further regression of SLL	none

**Figure 1:**
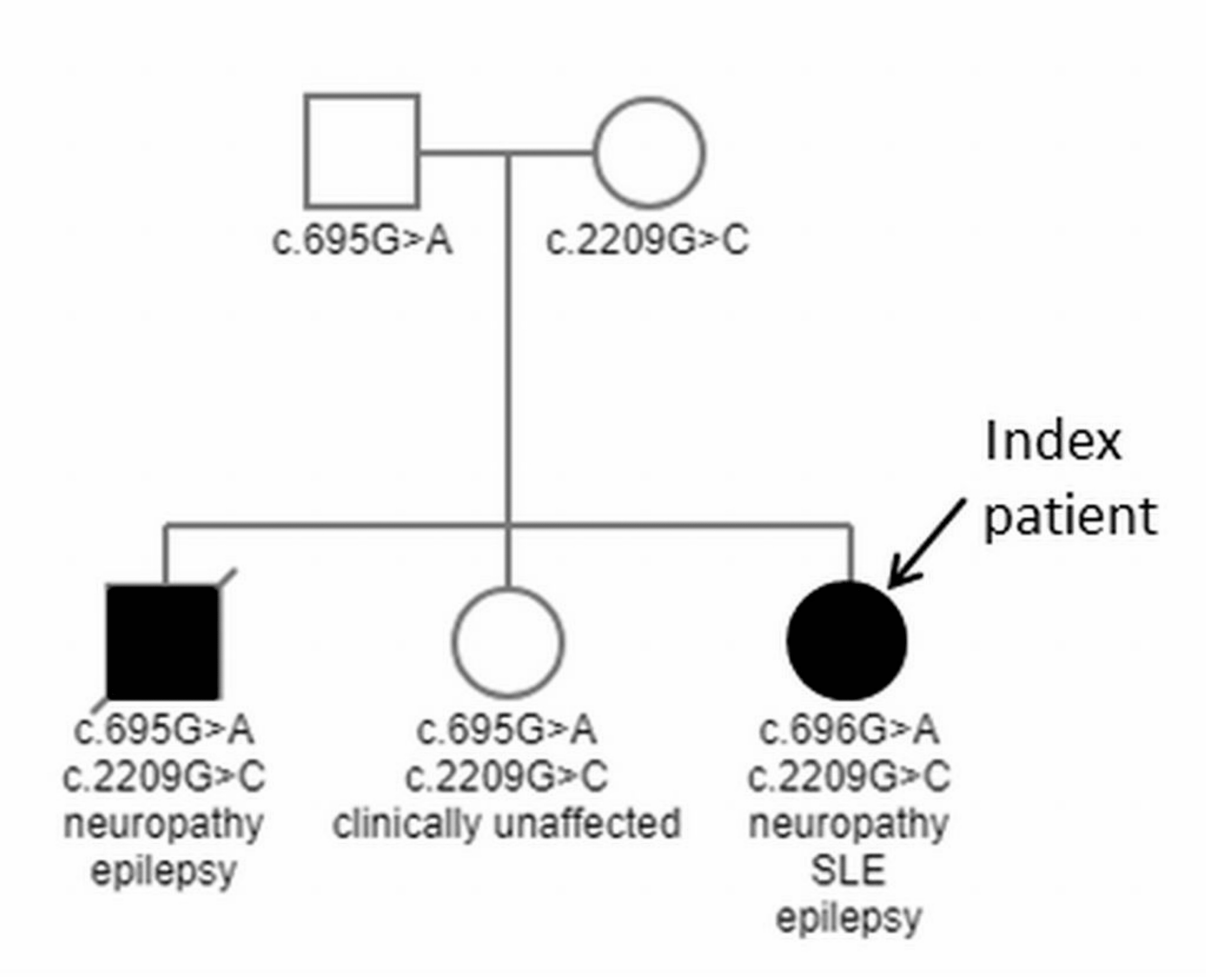
Pedigree with transmission of the variant c.695G>A from the mother and the variant c.2209G>C from the father to all three siblings, of whom only the index patient and her brother were clinically affected

The patient's medical history was negative for epilepsy. Family history was positive for a *POLG*-related disorder in the brother, who died of complications after prolonged status epilepticus at the age of 20 (Figure 1). In addition to epilepsy, he had shown phenotypic polyneuropathy from the age of 13, which is why a genetic investigation was initiated involving his parents and sisters, in which the variants c.695G>A in exon 3 and c.2209G>C in exon 13 of the *POLG* gene were detected in all three siblings (Figure 1). The c.695G>A variant was inherited from the father and the c.2209G>C variant from the mother (Figure 1). The c.695G>A variant resulted in the amino acid change p.Arg232His, rs113994093, and the c.2209G>C variant resulted in the amino acid change p.Gly737Arg. s121918054. The mother also carried the variant c.2171G>A (p.Gly724Glu, rs201524659) in exon 14 of the *TT-K2* gene and the father also carried the heterozygous variant c.2248G>A (p.Val750lle, rs148171058) in exon 14 of the *TRPV4-0* gene and the variant c.1550G>T (p. Gly517Val, rs61752783) in exon 8 of the *POLG* gene. The index patient’s 16-year-old sister also carried the two pathogenic *POLG* variants but was not yet phenotypically apparent.

**Figure 2:**
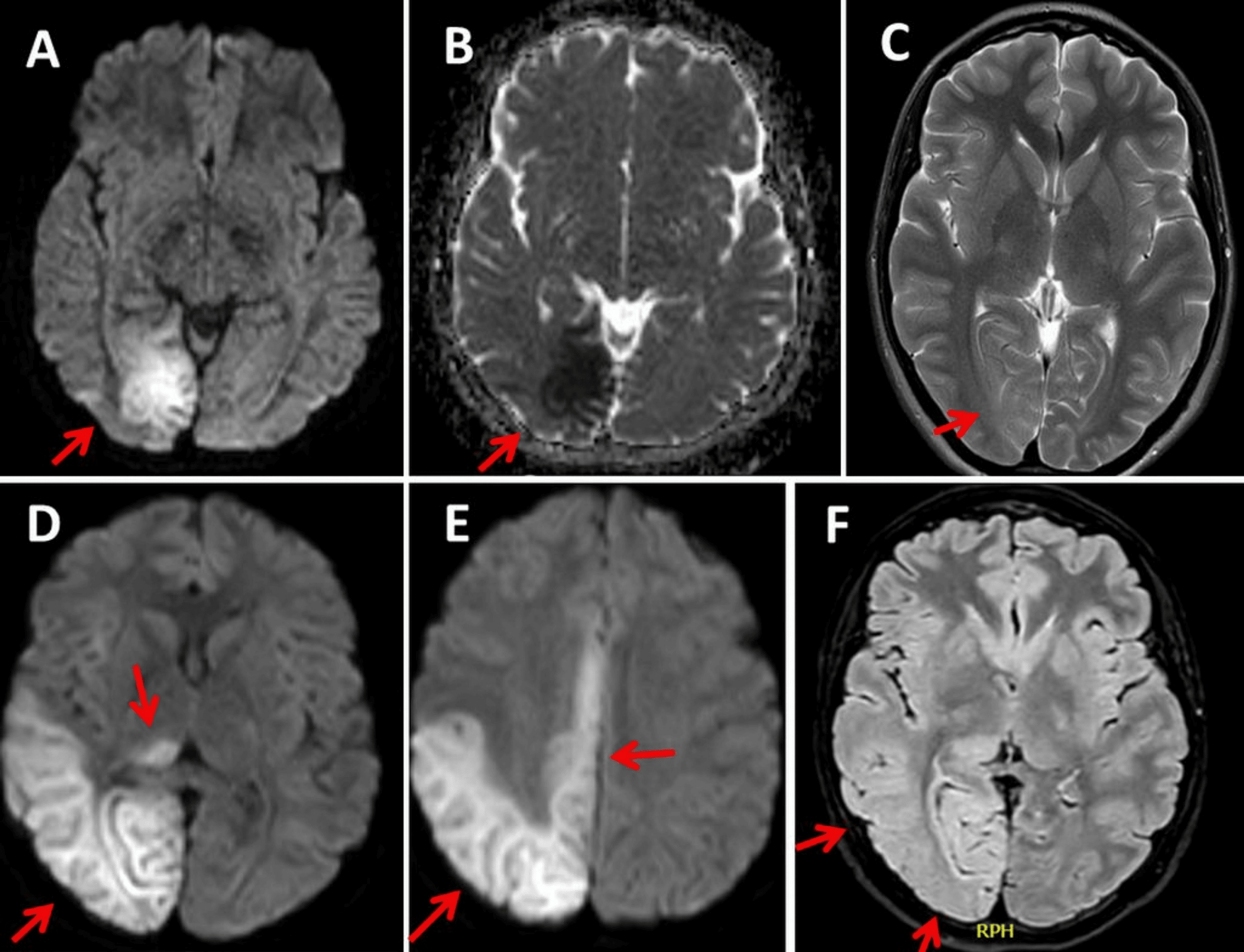
MRI of the brain at admission (Day 1) shows an occipital lesion that was hyperintense on T2/FLAIR (panel C) and DWI (panel A) and hypointense on ADC (panel B). The cerebral MRI on hd3 shows an enlargement of the right occipital lesion (panels D, E, F) FLAIR: fluid-attenuated inversion recovery; DWI: diffusion-weighted imaging; ADC: apparent diffusion coefficient

MRI of the brain on admission showed an occipital lesion that was hyperintense on T2/fluid-attenuated inversion recovery (FLAIR) and diffusion-weighted imaging (DWI) and hypointense on apparent diffusion coefficient (ADC) (Figure 2). EEG showed focal slowing in the right posterior region with interictal epileptiform discharges. Treatment with levetiracetam (LEV) 2000 mg/day was started.

**Figure 3:**
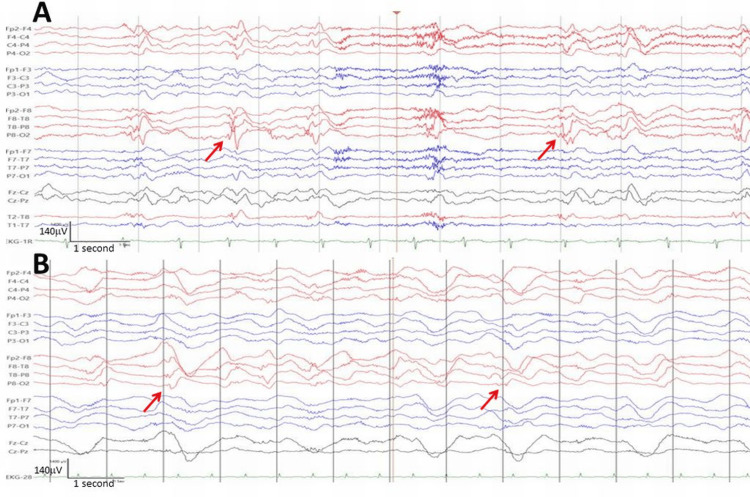
EEG recorded on hospital day 3 with LPDs or status epilepticus (panel A). LPDs were also recorded on hospital day 6 (panel B) (horizontal bar: 1s, vertical bar: 140V) LPD: lateral periodic discharge;

On HD 3, impaired consciousness and left-sided hemiparesis occurred. MRI showed an enlargement of the right occipital lesion (Figure 2). EEG showed lateral periodic discharges (LPDs) or status epilepticus (Figure 3). Antiseizure medication (ASM) was increased to LEV 3000 mg/day, lacosamide (LCM) 400 mg/day, and perampanel (PER) 12 mg/day. In addition to the ASM, the patient received a single dose of L-arginine (0.5 g/kg), which has since been continued at 0.1 g/kg.

**Figure 4:**
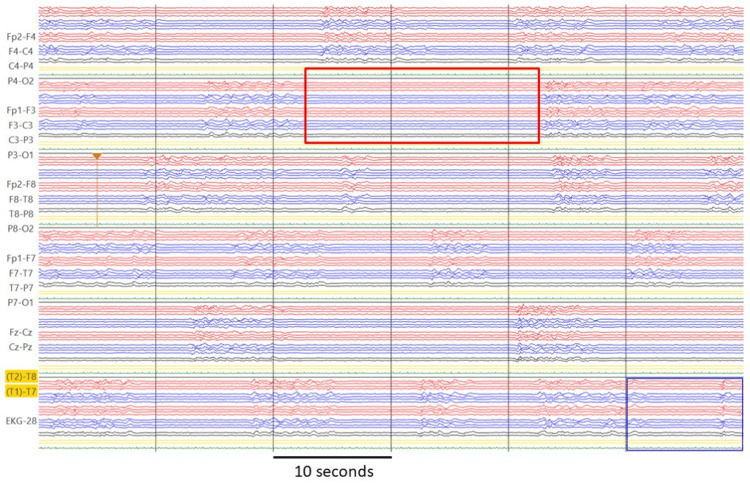
Monitor EEG recording on hospital day 7 under thiopental 250 mg/hour with BSP, with suppression up to 20 seconds (horizontal bar: 10s) BSP: burst suppression pattern

**Figure 5:**
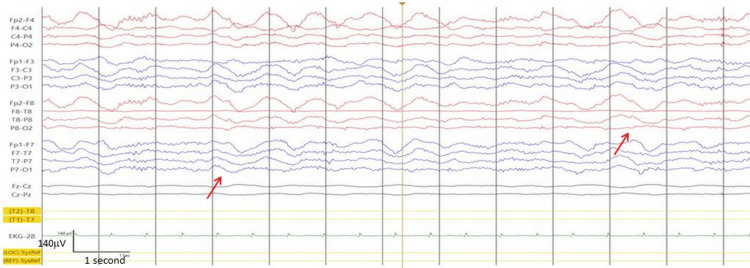
Routine EEG recording on hospital day 13 with delta activity after termination of status epilepticus (horizontal bar: 1s, vertical bar: 140V)

**Figure 6:**
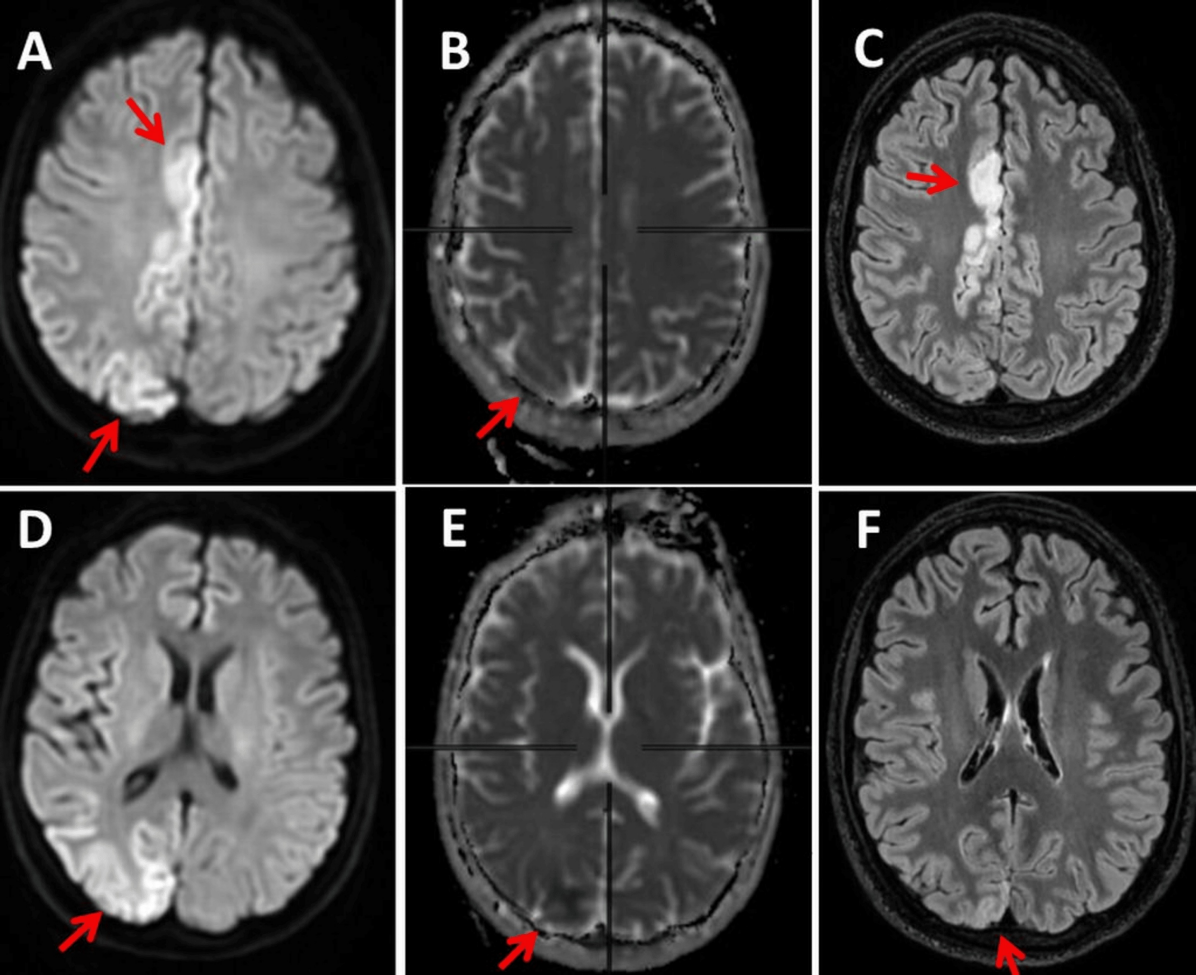
Cerebral MRI on hospital day 27, one day before extubation with regression of the SLL on DWI (panels A, D), ADC (panels B, E), and FLAIR (panels C, F) SLL: stroke-like lesion; DWI: diffusion-weighted imaging; ADC: apparent diffusion coefficient; FLAIR: fluid-attenuated inversion recovery

On HD 4, the EEG still showed LPDs, so ASM was extended to LCM 400 mg/day, PER 12 mg/day, and midazolam 45 mg/hour. Midazolam was increased to 60 mg/hour on HD 5. As the EEG was more or less unchanged on HD 6 (Figure 3), ASM was extended to intubation and anesthesia with thiopental (250 mg/hour) and ketamine (200 mg/hour), resulting in a burst suppression pattern (BSP) with burst durations of initially five to eight seconds and suppression of up to 20 seconds (Figure 4). In addition, methylprednisolone (1000 mg/day) was administered over four days (HD 6-9). On HD 7, suppression of up to 17 minutes was achieved with thiopental 250 mg/hour. On HD 8, thiopental was reduced to 200 mg/hour, on HD 9 to 150bmg/hour, and on HD 10 to 100 mg/hour. Riboflavin and coenzyme-Q were added. After discontinuation of thiopental on HD 12, continuous rhythmic delta activity was observed (Figure 5). MRI scan on HD 13 showed regression of the occipital lesion but a new DWI-hyperdense lesion in the right thalamus and cortical band, which is common in status epilepticus. Ketamine was reduced from 150 to 100 mg on HD 14 and 75 mg on HD 15 and discontinued on HD 17. Despite the reduction in anesthetics, the EEG still showed spikes on HD 15. Midazolam was reduced to 40 mg/hour on HD 18 and to 20 mg on HD 19 and discontinued on HD 21. The cerebral MRI on HD 27 showed further regression of the right occipital lesion (Figure 6).

**Figure 7:**
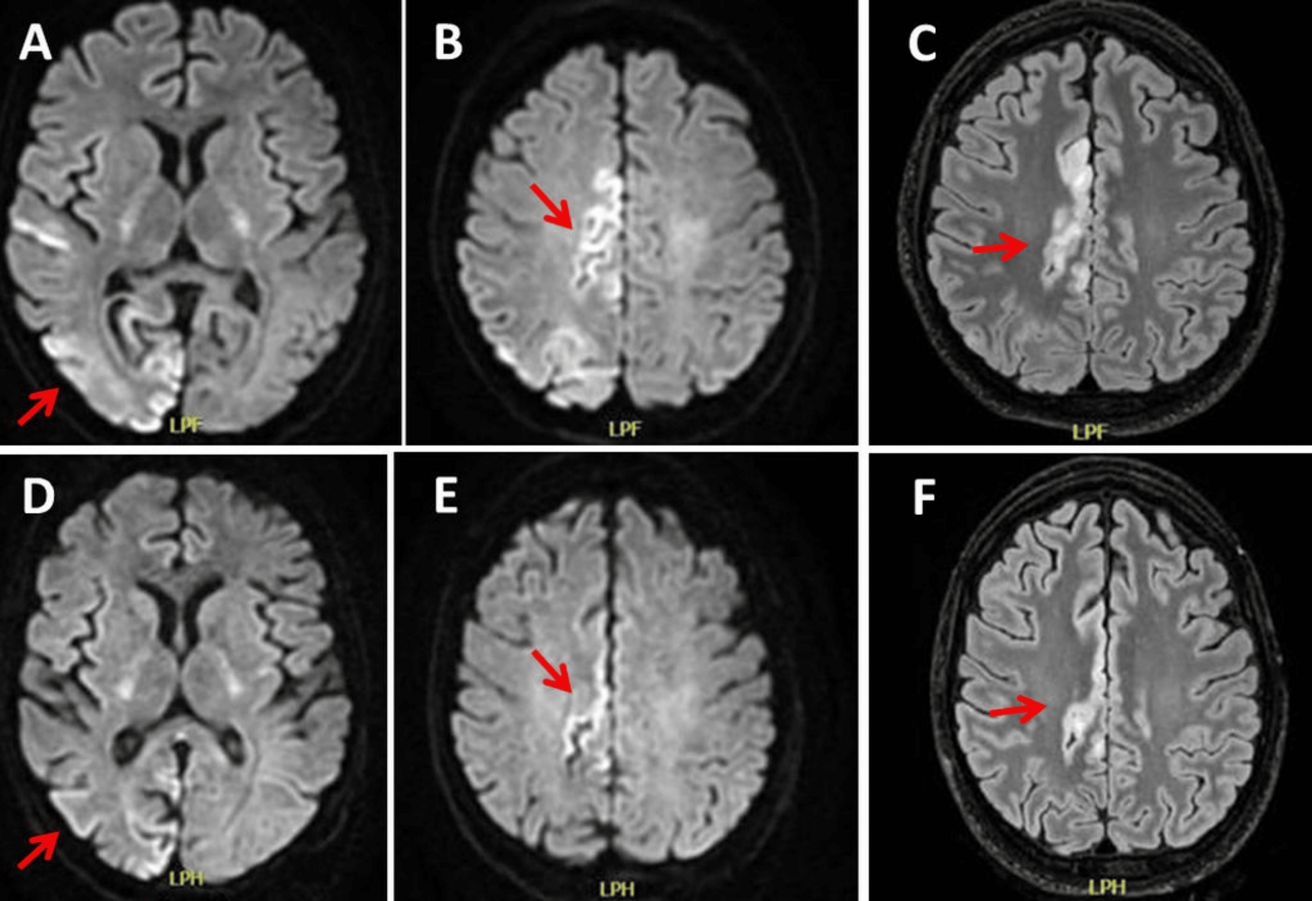
MRI of the brain on hospital day 27 (panels A-C) and on hospital day 52, showing further resolution of the SLL on T2 (panels D, E) and FLAIR (panel F) SLL: stroke-like lesion; FLAIR: fluid-attenuated inversion recovery

After extubation on HD 28 and tracheostomy, the patient developed recurrent focal motor status epilepticus with clonus of the right face and right shoulder, which could be stopped by the renewed administration of midazolam (1 mg/hour) and boluses. Nevertheless, the cerebral MRI on HD 31 showed a further regression of the T2/FLAIR, DWI, and ADC lesion in the right occipital lobe. Almost complete resolution of the SLL was observed on HD 52 (Figure 7). She is currently on medication with LEV 3000 mg/day, LCM 400 mg/day, PER 12mg/day, L-arginine 15g/day, vitamin Q10 (111.5 mg/day, vitamin B1 2100 mg/day, folinic acid 50mg/day, and fentanyl 75 mcg/hour.

## Discussion

This case is interesting in several respects. First, the patient carried a novel *POLG* variant in exon 3, which was found in compound heterozygosity with a rarely described *POLG* variant in exon 13. Second, the first clinical manifestation of the variant was an axonal sensorimotor neuropathy in the index patient and her brother. Third, the index patient suffered from SLE, which has rarely been reported in *POLG* variants. Fourth, the generalized status epilepticus could only be suppressed by anesthesia with thiopental, ketamine, and midazolam. Fifth, ASM possibly together with NO precursors and antioxidants had a favorable effect not only on seizure activity but also on SLL dynamics as an imaging correlate of SLE.

The variant c.2209G>C, which leads to the amino acid change p.Gly737Arg, has been reported previously by Phillips et al. [9], and classified as pathogenic or probably pathogenic [10]. In Phillips et al.'s case, their 20-year-old female patient showed slowed psychomotor development, delayed walking at three years of age, and delayed speech [9]. She subsequently developed progressive balance problems, spasticity, and pes equinus requiring Achilles tendon lengthening, tibialis posterior tendon transfer, the Jones procedure, and a walking aid from the age of 15. From that time, she also had achalasia with vomiting that required dilatations. At the age of 16, their patient presented with severe weakness of the ankle dorsiflexion, weakness of the intrinsic hand muscles, and moderate weakness of the hamstring muscles. There was pinprick hypoesthesia and pallhypoesthesia in the distal lower limbs. She was areflexive in the arms and legs. Nerve conduction studies (NCS) revealed severe axonal sensorimotor polyneuropathy. Their patient also carried two heterozygous variants in *POLG*, the variant c.2209G>C in exon 13 and the variant c.926G>A in exon 4 of POLG [9]. However, in contrast to the index patient, the patient reported by Phillips et al. did not manifest in the CNS.

The c.2209G>C variant was also reported in another patient, a 30-year-old man who presented phenotypically with tremors, dystonia, bradykinesia, and sensory disturbances since the age of 23 [11]. The tremor was associated with involuntary inversion of the feet and flexion of the right thumb, head posturing, and paresthesias in the right foot. Dystonia and tremor did not occur in the supine position, and there was a diurnal component that occurred only a few hours after waking. The patient was unable to walk in tandem but was able to walk on his heels and toes and support himself with his hand to maintain balance. NCS and somatosensory-evoked potentials (SSEPs) revealed evidence of moderate to severe generalized sensory length-dependent axonal polyneuropathy; cerebral and spinal MRI was not conclusive. A trial of low-dose levodopa/benserazide led to a significant improvement in tremor and dystonia; however, four years later, the patient also developed an onset of progressive external ophthalmoplegia, resting tremor in the right upper limb, occasional myoclonic jerks in the shoulder, arm, forearm, and distal fingers, appendicular cerebellar dysfunction, and mild bilateral foot drop. In addition to c.2209G>C, the patient carried the heterozygous variant c.3305A>C [11].

The reason why the phenotypic expression was heterogeneous in the index patient in the current report and the other two patients [9,11] remains speculative, but one reason could be that the second mutation besides the c.2209G>C variant was different in all three patients. The similarities between the three patients concerned the development of axonal polyneuropathy in all of them. However, the patient reported by Phillips et al. did not develop CNS manifestations [9], and the patient reported by Qiu et al. did not have SLEs [11]. However, SLE has also been reported in patients carrying variants other than those of the index patient. Cheldi et al. reported a 74-year-old man with SLE and myopathy due to the three heterozygous *POLG* variants c.752C>T in exon 3, c.1760C>T in exon 10, and c.3556G>C in exon 22 [12]. Surprisingly, the cerebral lesions found on imaging were interpreted as ischemic, but are more likely metabolic in nature given the multisystem nature of the disease and the genetic background. Martikainen et al. also reported SLE in a 26-year-old woman with headache, visual flashes, dysarthria, and generalized seizures [13]. EEG showed non-convulsive status epilepticus and cerebral MRI showed T2 hyperintensity in a parieto-occipital distribution consistent with SLL. Genetic testing revealed the c.2243G>C variant in *POLG*, and their patient benefited from phenytoin, oxcarbazepine, and LEV [13]. SLEs have also been reported in patients with AHS [14]. In a study of six *POLG* mutation carriers, all of them also had SLEs [15].

Seizures and status epilepticus are common manifestations of *POLG *variants. In a study of nine AHS patients by van Westrhenen et al., EEG recordings showed rhythmic high-amplitude deltas with superimposed (poly)spikes (RHADS) in all of them [16]. In six of the AHS patients, RHADS were present at first status epilepticus. Sometimes they appeared 5-10 weeks later and disappeared over time. RHADS were symptomatic in three AHS patients, and five AHS patients showed distinct ripples on the (poly)spikes of RHADS. In another study of five AHS patients by Wolf et al. with focal-clonic or complex-focal status epilepticus as the initial manifestation of the disease, three of them died 3-12 months after the onset of seizures [17]. In four children, the first EEG showed RHADS. Some of these patients did not respond adequately to established ASM and required more unusual therapy. In two women with AHS who manifested with seizures, these did not stop despite the use of multiple ASMs [18]. However, after the initiation of intravenous magnesium as ASM, clinical and neurophysiological improvement and rapid extubation of both patients were achieved. In general, there is no difference in the treatment of status epilepticus with and without SLE.

The pathophysiological consequences of *POLG* mutations are not always clear, but it is known that *POLG* mutations impair mtDNA synthesis and consequently lead to depletion and impaired repopulation of mtDNA in intact cells. A known pathophysiological consequence of *POLG* mutations is the upregulation of Notch and Janus Kinase-Signal Transducers and Activators of Transcription (JAK-STAT) signaling pathways. These signaling pathways are crucial for neuronal development. The upregulation of Notch signaling pathways also leads to stem cell activation and increases oxidative stress. Oxidative stress can be exacerbated by increased NO production due to endothelial and vascular dysfunction in cells with *POLG* mutations. Increased oxidative stress and increased NO production may be the reason why antioxidants and NO precursors are beneficial in patients with MID. Whether the use of NO precursors and antioxidants actually had a beneficial effect not only on seizure activity but also on the manifestations of SLL in the index patient remains speculative; however, since such a beneficial effect has been previously reported [19,20], it is conceivable that these drugs also favorably influenced the dynamics and manifestations of SLL. Regardless of these potential mechanisms and treatment effects, the most effective treatment for SLLs remains ASM when SLLs present with seizures.

The limitations of this case study were that biochemical assays were not performed, mtDNA copy number was not measured, and mtDNA was not sequenced to assess whether the *POLG* mutations caused secondary single or multiple mtDNA deletions.

## Conclusions

This case demonstrates that pathogenic *POLG* variants may initially manifest phenotypically with polyneuropathy, *POLG* variants may manifest with SLE, which occurs before the onset of seizures, status epilepticus due to *POLG* variants may require thiopental anesthesia, and LEV, LCM, and PER may be beneficial for seizure activity in these patients.
